# Cognitive and White-Matter Compartment Models Reveal Selective Relations between Corticospinal Tract Microstructure and Simple Reaction Time

**DOI:** 10.1523/JNEUROSCI.2954-18.2019

**Published:** 2019-07-24

**Authors:** Esin Karahan, Alison G. Costigan, Kim S. Graham, Andrew D. Lawrence, Jiaxiang Zhang

**Affiliations:** Cardiff University Brain Research Imaging Centre, School of Psychology, Cardiff University, Cardiff CF24 4HQ, United Kingdom

**Keywords:** along tract analysis, cognitive model, microstructure, NODDI, non-decision time, simple reaction time

## Abstract

The speed of motor reaction to an external stimulus varies substantially between individuals and is slowed in aging. However, the neuroanatomical origins of interindividual variability in reaction time (RT) remain unclear. Here, we combined a cognitive model of RT and a biophysical compartment model of diffusion-weighted MRI (DWI) to characterize the relationship between RT and microstructure of the corticospinal tract (CST) and the optic radiation (OR), the primary motor output and visual input pathways associated with visual-motor responses. We fitted an accumulator model of RT to 46 female human participants' behavioral performance in a simple reaction time task. The non-decision time parameter (*T*_er_) derived from the model was used to account for the latencies of stimulus encoding and action initiation. From multi-shell DWI data, we quantified tissue microstructure of the CST and OR with the neurite orientation dispersion and density imaging (NODDI) model as well as the conventional diffusion tensor imaging model. Using novel skeletonization and segmentation approaches, we showed that DWI-based microstructure metrics varied substantially along CST and OR. The *T*_er_ of individual participants was negatively correlated with the NODDI measure of the neurite density in the bilateral superior CST. Further, we found no significant correlation between the microstructural measures and mean RT. Thus, our findings suggest a link between interindividual differences in sensorimotor speed and selective microstructural properties in white-matter tracts.

**SIGNIFICANCE STATEMENT** How does our brain structure contribute to our speed to react? Here, we provided anatomically specific evidence that interindividual differences in response speed is associated with white-matter microstructure. Using a cognitive model of reaction time (RT), we estimated the non-decision time, as an index of the latencies of stimulus encoding and action initiation, during a simple reaction time task. Using an advanced microstructural model for diffusion MRI, we estimated the tissue properties and their variations along the corticospinal tract and optic radiation. We found significant location-specific correlations between the microstructural measures and the model-derived parameter of non-decision time but not mean RT. These results highlight the neuroanatomical signature of interindividual variability in response speed along the sensorimotor pathways.

## Introduction

Voluntary response to external stimuli is a hallmark of cognitive control that encompasses perceptual, decision and motor processes. The reaction time (RT), measured as the latency between a preparatory stimulus and a predefined action, varies substantially across individuals (Jensen, 2006), changes during development (Dykiert et al., 2012), aging (Woods et al., 2015) and neurodegeneration (Gorus et al., 2008), and has implications for mortality (Der and Deary, 2018). RT has also been identified as a marker of mental processing speed (Ho et al., 1988; Sheppard and Vernon, 2008), a heritable trait relating to intelligence (Vernon, 1989; Rabbitt, 1996).

Individual differences in RT have been regarded as reflections of a “primitive” neurophysiological characteristic (Rabbitt et al., 2001), and the potential of RT as a behavioral phenotype necessitates understanding its microstructural underpinnings (Turken et al., 2008; Penke et al., 2010). In humans, diffusion tensor imaging (DTI) is commonly used to estimate tissue microstructure from diffusion-weighted MRI (DWI; Basser and Pierpaoli, 1996), which is sensitive to the degree of anisotropic water diffusion because of cellular structures. The DTI measures of several white-matter pathways have been shown to correlate with RT, both in adults (Tuch et al., 2005; Turken et al., 2008; Johnson et al., 2015) and children (Madsen et al., 2011; Tamnes et al., 2012; Scantlebury et al., 2014), possibly because of the difference in experiments and cohorts across studies. One hypothesis of these structural–functional correlations is that the interindividual variability in RT is due to variations in tissue microstructure, such as axon diameter or myelination that affects the nerve conduction velocity (Waxman, 1980; Fields, 2008), which in turn affects spike timing and oscillatory coupling over long-range connections (Pajevic et al., 2014) that are necessary for motor control (Baker et al., 2003; Pogosyan et al., 2009) and RT (Chevalier et al., 2015; Chopra et al., 2018).

However, research on the relationship between RT and DTI raises two unresolved issues. First, RT, even in simple tasks, is a hybrid measure of multiple intermixed cognitive processes (Gold and Shadlen, 2007; Forstmann et al., 2016; Ratcliff et al., 2016). Second, the conventional DTI model cannot distinguish microstructural properties between intracellular and extracellular space, because it assumes a single tissue compartment (Beaulieu, 2002).

This study addressed these questions by combining a cognitive model of RT and a biophysical compartment model of the DWI signal. We used the cognitive model (Brown and Heathcote, 2008) to decompose a non-decision time measure from individual participant's RT distribution in a simple reaction time task, which accounts for the latencies of stimulus encoding and action initiation (Lo and Wang, 2006; Donkin et al., 2011). DWI data were analyzed with the neurite orientation dispersion and density imaging (NODDI) model (H. Zhang et al., 2012). NODDI estimates separately the isotropic and anisotropic diffusion in multiple compartments, allowing two specific measures of tissue microstructure: the neurite density index (NDI) as the intracellular volume fraction and the orientation dispersion index (ODI) explaining the bending or fanning of axon orientations.

Furthermore, using probabilistic tractography and volume skeletonization, we developed a new method to quantify the changes of microstructural metrics along fiber tracts. We focused on two a priori tracts according to the functional anatomy of voluntary movement and visual processing that are relevant to the simple reaction time task. The first was the corticospinal tract (CST), a major output pathway carrying motor impulses from the giant pyramidal cells in the motor area to the midbrain (Lemon, 2008). The second was the optic radiation (OR), the prominent white-matter relay in the visual system, transmitting information from the lateral geniculate nucleus (LGN) to V1 (Garey and Powell, 1971; Ebeling and Reulen, 1988).

Our results demonstrated that microstructural metrics varied substantially along CST and OR. Higher neurite density in bilateral superior CST was associated with faster non-decision time across participants, but not with the mean RT. These findings suggested microstructural-specific influence of interindividual variability in subcomponents of the action decision process.

## Materials and Methods

### 

#### 

##### Participants.

Forty-six healthy participants were recruited from the Cardiff University School of Psychology participant panel (all females, age range 19–24 years; mean age 20.8 years). Participants had normal or corrected-to-normal vision, and none reported a history of neurological or psychiatric illness. This study was approved by the Cardiff University School of Psychology Research Ethics Committee. Written informed consent was obtained from all participants.

##### Experimental design.

**Figure 1.**
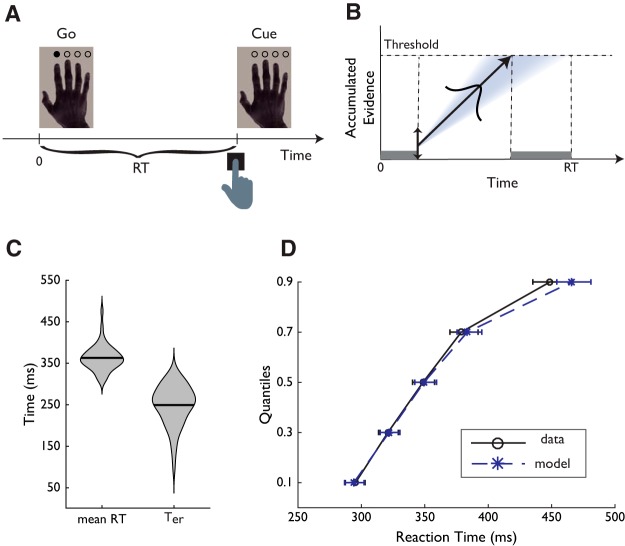
***A***, Experimental paradigm of the SRT task. Participants were instructed to respond when a filled circle appeared over the index finger in the hand picture. ***B***, Exemplar time course of the LBA model. The LBA assumes that accumulated evidence for an action decision is accumulated linearly over time, and a decision is made once the accumulated evidence reaches a threshold. In each trial, the starting point of the accumulation process is sampled from a uniform distribution. The rate of accumulation is sampled from a Gaussian. The model predicted RT includes the duration of the accumulation process and a non-decision time (*T*_er_), that accounts for the latencies of non-decision processes such as stimulus encoding and action initiation, which are shown as shaded area. ***C***, Violin plots (mean and density) of the mean RT and *T*_er_ across participants. ***D***, The fit of the LBA model to RT quantiles in the SRT task. Error bars denote the 95% confidence intervals.

Participants performed a visually paced simple reaction time (SRT) task adapted from previous studies (J. Zhang et al., 2012; Shafto et al., 2014). The task was conducted in a behavioral testing room. The participants were presented with an image of a right hand on a 24-inch LED monitor with 1920 × 1080 screen resolution (ASUS VG248QE) and pressed the spacebar on the keyboard button with their right index finger. Four transparent circles superimposed above the four fingers in the hand image to serve as task cues (Fig. 1*A*). On each trial, the task cue above the index finger in the image turned to an opaque circle, indicating the start of the trial, and the participants were instructed to respond as quickly as possible. After participants' response or after a maximum of 3 s response window, the opaque task cue extinguished and changed back to a transparent circle. Visual stimuli were presented by using Microsoft Visual Basic 5.0.

After a short practice, the participants performed 50 trials of the SRT task. To discourage proactive response strategies, the intertrial interval randomly varied across trials with a skewed distribution (minimum 1800 ms, maximum 6800 ms, and mean 3700 ms). RT was measured as the latency between task cue onset and button press. Trials with RT <150 ms or >1500 ms were excluded from further analysis (0.65% of the total number of trials across all participants). Mean RT was then calculated as the behavioral dependent measure for each participant.

##### Accumulator model of simple actions and parameter estimation.

We further analyzed the RT data using a cognitive model of RT, the linear ballistic accumulator (LBA) model (Brown and Heathcote, 2008). The LBA model is a simplified implementation of a large family of sequential sampling models (Ratcliff and Smith, 2004; Bogacz et al., 2006; Gold and Shadlen, 2007; J. Zhang, 2012) and has been used to study the cognitive processes underlying decision making (Ho et al., 2009; Forstmann et al., 2011), action selection (J. Zhang et al., 2012), and action inhibition (Sebastian et al., 2018).

In the present SRT task, there was only one possible action. We assumed that the process is governed by a linear evidence accumulation process (Fig. 1*B*), from a randomly sampled starting point to a decision threshold *B* (for similar approaches, Ratcliff and Van Dongen, 2011; Schurger et al., 2012). The speed of evidence accumulation varies across trials as a Gaussian random variable with a mean μ and SD σ. The model-predicted RT is given by the duration of the accumulation process to reach a decision threshold *B*, plus a constant non-decision time *T*_er_. The non-decision time *T*_er_ does not relate to evidence accumulation, but accounts for the latency of other processes including motor response initiation and stimulus encoding (Gold and Shadlen, 2007; Brown and Heathcote, 2008).

We fitted the LBA model to the RT distribution from the SRT task using a minimization procedure validated in previous studies of RT modeling (Bogacz and Cohen, 2004; Bogacz et al., 2006; Boucher et al., 2007; Dean et al., 2011; J. Zhang et al., 2012, 2016). For each participant, the observed RT distribution was binned into the 0.1, 0.3, 0.5, 0.7, and 0.9 quantiles (Ratcliff and Smith, 2004), and the model prediction of the five RT quantiles were estimated from 100,000 numerical simulations. The starting point variability was fixed at 0.5 as the scaling parameter (Brown and Heathcote, 2008; Donkin et al., 2009). The model parameters (*T*_er_, *B*, μ, and σ) were determined by minimizing the likelihood ratio χ^2^ statistic between the observed and predicted RT distributions using the Simplex search algorithm (Nelder and Mead, 1965). To optimize the chance of locating the optimal model parameters, the minimization procedure started with a set of initial parameter values. Each initial parameter set was chosen from 100 randomly generated values that produced the best fit. The entire minimization procedure was then repeated 20 iterations to identify the best-fitting model parameters. The two time-dependent measures, the non-decision time *T*_er_ and the mean RT, were then associated with microstructural metrics from diffusion MRI.

##### MRI data acquisition.

Whole-brain two-shell DWI data were acquired using a Siemens 3T Prisma MRI scanner and a 32-channel receiver head coil (Siemens Medical Systems) at the Cardiff University Brain Research Imaging Centre with single-shot spin-echo echoplanar imaging pulse sequence (echo time 67 ms, repetition time 9400 ms, field-of-view 256 × 256 mm, acquisition matrix size 128 × 128, voxel size 2 × 2 × 2 mm). Diffusion sensitizing gradients were applied in 30 isotropic directions at a *b* value of 1200 s/mm^2^ and in 60 isotropic directions at a *b* value of 2400 s/mm^2^. Six images with no diffusion weighting (*b* = 0 s/mm^2^) were also acquired. Participants also underwent high-resolution T1-weighted magnetization prepared rapid gradient echo scanning (MPRAGE; echo time: 3.06 ms; repetition time: 2250 ms sequence, flip angle: 9°, field-of-view: = 256 × 256 mm, acquisition matrix: 256 × 256, voxel size: 1 × 1 × 1 mm).

##### DWI data processing and modeling.

DWI data were converted from DICOM to NIfTI format using dcm2nii (RRID:SCR_014099; http://www.nitrc.org/projects/dcm2nii). The images were skull-stripped, and corrected for eddy currents and head motion using FSL BET and eddy_correct functions (FSL 5.0.9, http://www.fmrib.ox.ac.uk/fsl). DWI data from both shells were registered to the first non-diffusion (*b* = 0 s/mm^2^) volume.

**Figure 2.**
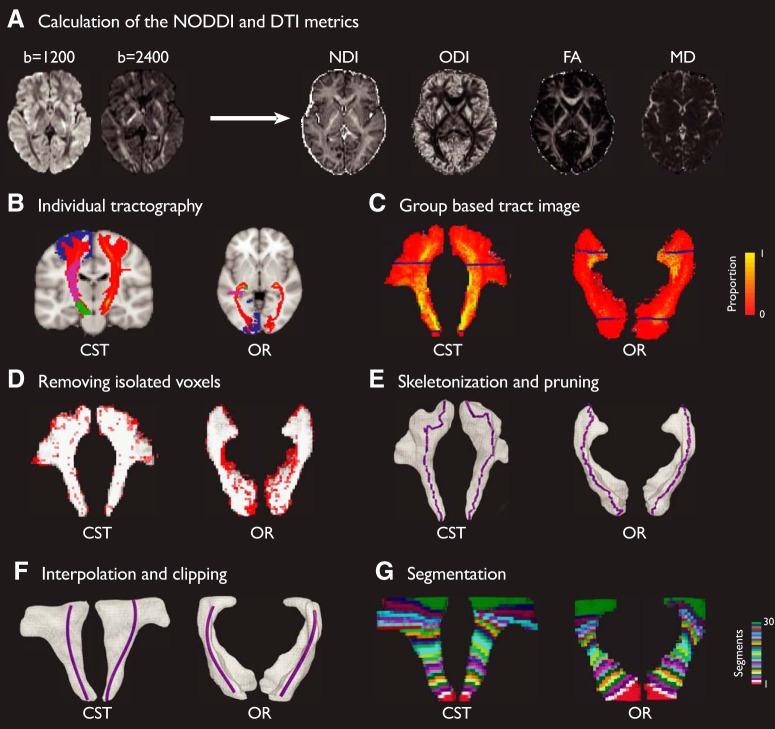
Analysis pipeline of along-tract microstructural metrics. ***A***, After preprocessing, whole-brain voxelwise NODDI (NDI and ODI) and DTI (FA and MD) metrics were calculated from the two-shell DWI data. ***B***, Probabilistic tractography of CST and OR were calculated and thresholded in individual participant's native space. Seed (green), waypoint (purple), and termination masks (blue) used for the tractography are overlaid for both tracts. ***C***, Individual tractography results of CST and OR were normalized to the MNI space and united to obtain group-based tract images. The voxel intensity in the group-based tract images denotes the proportion of overlapping across participants. ***D***, Isolated voxels (red) with no second-order neighbors were removed from the group-based tract volumes. ***E***, Three-dimensional group-based tract volume image was skeletonized using thinning algorithms. Subsidiary branches of the skeleton were trimmed. ***F***, The main skeleton was smoothed with cubic spline interpolation. The central portion of the group-based tract volume (between the two blue planes shown in ***C***) and the skeleton were clipped for further processing. ***G***, The skeletons of the CST and OR were divided into 30 segments with equal lengths and 20% overlap between adjacent segments. Voxels in each group-based tract volume were assigned to the nearest segment based on Euclidean distance, resulting in 30 equidistant sub-volumes along the principal skeleton. Each sub-volume was transformed back to the individual's native space to calculate microstructural metrics along tracts.

After preprocessing, diffusion tensors were fitted to the DWI data using DTIFIT in FSL for each shell (*b* = 1200 s/mm^2^ and *b* = 2400 s/mm^2^). Local fiber orientation distributions in each voxel were estimated by using the ball and stick model for multiple shells (Behrens et al., 2003; Jbabdi et al., 2012). For each voxel, we calculated two DTI measures, fractional anisotropy (FA) and mean diffusivity (MD) by using the *b* = 1200 s/mm^2^ shell data (Fig. 2*A*). FA is a scalar ranging from 0 to 1 that quantifies the coherence of water diffusion, with 0 indicating low coherence and 1 indicating high coherence, whereas MD measures the average rate of water diffusion, with higher rates indicating fewer boundaries for water diffusion (Basser and Pierpaoli, 1996). Only data with *b* = 1200 s/mm^2^ was used for the calculation of FA and MD, because the DTI model assumes only hindered diffusion in the extra-axonal space which is more sensitive to lower *b* values (Le Bihan et al., 2001).

The same preprocessed DWI data were fitted to the NODDI model using the NODDI MATLAB Toolbox v1.0.1 (https://www.nitrc.org/projects/noddi_toolbox). The NODDI model contains three tissue compartments: intracellular space, extracellular space, and CSF. The intracellular estimation uses the stick model to capture the restricted diffusion perpendicular to neurites and unhindered diffusion along them. The extracellular compartment models the hindered diffusion of water molecules by Gaussian anisotropic diffusion with parallel and perpendicular diffusivities. The CSF compartment is modeled as isotropic Gaussian diffusion to minimize the confounding effect of CSF contamination (H. Zhang et al., 2012). From the model-derived intracellular and extracellular compartments, we calculated two voxelwise NODDI measures, NDI, and ODI (Fig. 2*A*). NDI represents the volume fraction of the intracellular compartment that contains the axons and dendrites, and ODI quantifies the angular variation in neurite orientation. Neurite orientation distribution was parametrized with the Watson distribution by using the “WatsonSHStickTortIsoV_B0” model.

The high-resolution T1-weighted MPRAGE image was linearly coregistered to the native DWI space using mutual information with 6 degrees of freedom. The coregistered MPRAGE image was segmented and normalized to the Montreal Neurological Institute (MNI) standard template by linear and nonlinear deformations using FSL. The forward and inverse deformation fields between the native DWI space and the MNI template space were used for subsequent tractography and along-tract analysis.

##### Tractography.

For each participant, we conducted probabilistic tractography to reconstruct bilateral CST, OR, and dorsal cingulum bundle (CB; the latter as a comparison tract) in the individual's native space using FMRIB's Diffusion Toolbox (Behrens et al., 2007). For all tracts, we used multiple regions-of-interest (ROIs) from the Jülich histological atlas (Bürgel et al., 2006) and John Hopkins University (JHU) DTI-based white-matter atlases (Hua et al., 2008) to define seed masks, target masks, waypoints, and exclusion masks.

Each tract was reconstructed by sampling 5000 streamlines per voxel with 0.5 mm step length, 0.2 curvature threshold, 0.1 fiber threshold, and 3 mm minimum streamline length. The probabilistic tractography procedure generates an image in which the intensity of each voxel is the ratio of the number of streamlines that pass through that voxel over the total number of streamlines generated in the seed voxels (Fig. 2*B*). We thresholded the probabilistic tractography outputs at 5 × 10^−5^ to discard false-positives (Rilling et al., 2008).

For the CST, we followed a previous method (Y. Zhang et al., 2010) to use the ROIs from the JHU atlases. The tractography was seeded from the cerebral peduncle (CP) and extended through the posterior limb of interior capsule and superior corona radiata (SCR). The target mask was the intersection of the precentral gyrus from the JHU atlas with the individual gray matter images from MPRAGE segmentation (Fig. 2*B*). The target mask was dilated with a 2 mm disk-shaped kernel using the Image Processing Toolbox in MATLAB to account for anatomical variability across individuals (Clatworthy et al., 2010). Exclusion masks included the contralateral hemisphere, anterior limb of the internal capsule, the retrolenticular part of the internal capsule, the pontine crossing tract, and the inferior and superior cerebellar peduncle.

For the OR, the tractography was seeded from the LGN in each hemisphere, and a central slice of OR defined in the Jülich histological atlas was used as a waypoint mask to avoid the fibers that transverse Meyer's loop (Clatworthy et al., 2010). The target mask was the primary visual cortex, jointly defined by the Jülich histological atlas and the individual gray matter mask from MPRAGE segmentation. This target mask was further dilated with a 2 mm disk-shaped kernel to account for the individual anatomical variability (Fig. 2*B*). Exclusion masks included the anterior boundary of the OR in the Jülich atlas and the contralateral hemisphere (Clatworthy et al., 2010).

For the CB, we used the cingulum defined in the Jülich histological atlas to localize the seed and target masks. The tractography was seeded from the anterior section of the cingulum and a central slice of the same image was used as a waypoint mask. The target mask was located at the posterior section of the cingulum.

**Figure 3.**
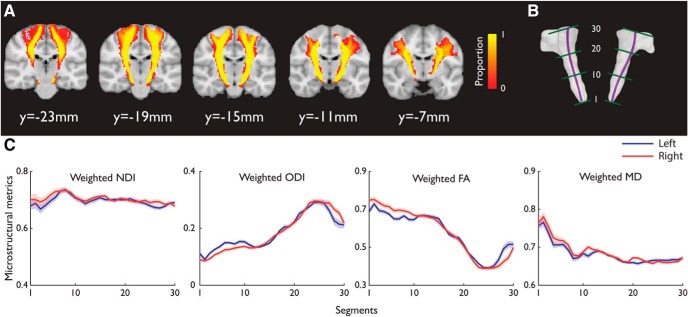
***A***, Group-based image of the CST. The voxel intensity denotes the proportion of overlapping across participants. ***B***, Visualization of the CST with the skeleton and the approximate locations of segments 1, 10, 20, and 30. ***C***, The means of NDI, ODI, MD, and FA profiles of the left and right CST across participants. Shaded areas represent 95% CI. The segments 1–30 were from CP to SCR as in ***B***.

**Figure 4.**
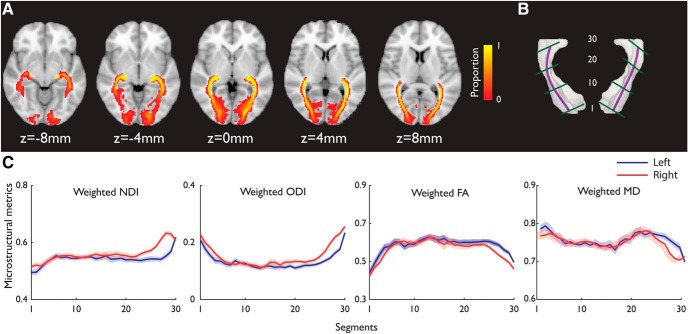
***A***, Group-based image of the OR. The voxel intensity denotes the proportion of overlapping across participants. ***B***, Visualization of the OR with the skeleton and the approximate locations of segments 1, 10, 20, and 30. ***C***, The means of NDI, ODI, MD, and FA profiles of the left and right OR across participants. Shaded areas represent 95% CI. The segments 1–30 were from V1 to posterior LGN as in ***B***.

After tractography, we generated a group-based representation of each tract in the MNI space across all participants (Figs. 2*C*, 3*A*, 4*A*). The individual thresholded tractography results were binarized following the transformation to the MNI space by applying the forward deformation field from MPRAGE normalization. A group-based tract image was calculated from the union of all participants' normalized tracts.

##### Tract skeletonization.

Similar to previous studies (Yeatman et al., 2012), we performed tractography-based skeletonization for subsequent along-tract analysis in native space. This procedure is different from FA-based skeletonization methods, which are typically used for alignment-invariant tract representation for voxelwise analysis (Smith et al., 2006).

For each tract, we generated a skeletal profile of the group tract images using in-house scripts and the volume skeleton toolbox in MATLAB (Fig. 2*D*; Cornea et al., 2007). First, the group tract image was binarized. The isolated voxels with no second order neighbors were removed. Holes and discontinuities in the tract image volume were removed by dilating the whole tract volume with a 2 mm disk-shaped kernel. Second, we applied the thinning algorithm to estimate the skeleton of the cleaned tract images (Cornea et al., 2007). The thinning algorithm estimates the curve-skeleton of a three-dimensional object by iteratively removing simple voxels from the surface boundary. Simple voxels are defined as the ones whose removal would not change the topology of the volume. This operation was repeated until no simple voxels remained. To find the main curvature of the tract, branches along the main trunk of the skeleton were removed. A cubic spline interpolation was fitted to the principal trunk of the skeleton to obtain a smoothed three-dimensional skeleton of the tract. Third, similar to previous approaches (Yeatman et al., 2012), we focused on the central portion of the tracts, where fiber bundle morphology is most consistent across individuals. Therefore, we clipped the group-based CST image and the skeleton to the portion between the cerebral peduncle and the superior corona radiata, and we clipped the group-based OR image and the skeleton to the portion closer to anterior regions of V1 and posterior regions of LGN.

##### Microstructural measures along tracts.

To quantify microstructural metrics and their changes along tracts, we divided the spline interpolation of each group-based tract skeleton into 30 segments with equal length and 20% overlap between adjacent segments, although our main results were not dependent on the exact number of segments. All the voxels in the group-based tract image were assigned to the corresponding segment based on the closest Euclidean distance. This procedure was repeated for all the tracts in the left and right hemispheres, resulting in 30 equidistant sub-volumes along the principal skeleton of each group-based tract image (Fig. 2*E*). Because adjacent segments were overlapping, the labeled sub-volumes also had overlapping voxels. This interpolation ensured the along-tract microstructural measures were less affected by segment boundaries (Yeatman et al., 2012).

All the sub-volumes were transformed back to the individual's native space to reconstruct participant specific sub-volumes along tracts. For each participant, a voxel in a sub-volume was included in the calculation of individual microstructural metrics only if: (1) it was in the individual white-matter mask from the MPRAGE segmentation, (2) it had a FA value >0.2 from DTI modeling, and (3) it had a suprathreshold probability of connection from the tractography results.

This approach enables quantification of microstructural measures at multiple locations (i.e., segments) along the principal trajectory of a white-matter tract, reducing three-dimensional volumetric data to one-dimensional tract profiles. There are two advantages to quantify microstructural measures at the segment level rather than the voxel level. First, it enables estimation of the variation of microstructure along tract trajectories, which are otherwise not directly observable in voxelwise analysis or when averaging over the entire tract. Second, the combination of group-level segments and individual-level constraints balances the consistency and variability across participants when making inferences along tracts.

For each participant, the four microstructural metrics (NDI, ODI and FA, MD) at each sub-volume of each tract were calculated as a weighted average of the metrics from all voxels within the sub-volume *M*:

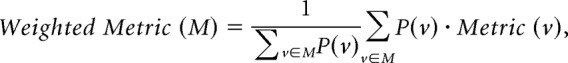
 where the weight *P*(*v*) for voxel *v* is the strength of connection in *v* estimated from probabilistic tractography.

##### Statistical analysis.

To examine the relationships between RT and *T*_er_, we used both frequentist and Bayesian Pearson's correlation tests across participants (JASP team, 2018). We used repeated-measures ANOVA with two factors of track segments and hemispheres to examine the change of microstructural measures along tracts and across hemispheres. Greenhouse–Geisser correction was applied where Mauchly's sphericity test indicated that the assumption of sphericity was violated.

For each sub-volume along a tract, we used general linear models to associate the weighted average of microstructural metrics to individual mean RT and *T*_er_ from the behavioral data. Because of the age-related changes in DWI signal (Yeatman et al., 2014; Cox et al., 2016), we included age as a nuisance variable in all the models. To correct for multiple comparisons from the 30 overlapping segments along each tract and 4 microstructural metrics (NDI, ODI, FA, and MD), we used threshold-free cluster enhancement (TFCE) in FSL PALM toolbox with 10,000 permutations to control familywise error at the cluster level in each tract and across multiple microstructural metrics (Smith and Nichols, 2009; Winkler et al., 2014).

##### Software accessibility.

The algorithms for tract skeletonization, tract segmentation and other analyses used in the current study is open-source and freely available online (https://github.com/esinkarahan/ATA).

## Results

We examined whether interindividual differences in brain microstructure would be associated with the reaction time of simple motor actions. Participants were required to perform a right-hand SRT task (Fig. 1*A*) to measure their mean RT (Fig. 1*C*). Next, we used the LBA model (Brown and Heathcote, 2008) to quantify the non-decision time *T*_er_ during simple actions (Fig. 1*B*), a model-derived parameter to account for the latency of motor initiation and stimulus encoding (Lo and Wang, 2006; Cavanagh et al., 2011; Donkin et al., 2011). The two behavioral measures were then correlated with microstructural metrics from NODDI and DTI models in the primary motor and visual pathways: the CST and OR, together with a comparison tract, the CB.

### Behavioral results

The mean RT across 46 participants was 363.02 ± 30.832 ms (SD) and the mean *T*_er_ was 249.238 ± 48.227 ms (SD; Fig. 1*C*). The LBA model provided an adequate fit to the observed RT distributions (Fig. 1*D*). We used frequentist and Bayesian Pearson's correlation to examine the intercorrelation between behavioral measures. There was no significant correlation between mean RT and *T*_er_ [*r* = 0.114, *p* = 0.45, 95% CI = (−0.182, 0.392), BF_10_ = 0.243], and no correlations between the behavioral measures and age (RT: *r* = 0.074, *p* = 0.624, 95% CI = (−0.221, 0.357), BF_10_ = 0.206; *T_er_*: *r* = 0.062, *p* = 0.683, 95% CI = (−0.233, 0.346), BF_10_ = 0.199].

### Tractography and along-tract microstructural metrics

We used a ROI-based probabilistic tractography approach to reconstruct four tracts (left CST, right CST, left OR, and right OR) in the individual's native space and obtained group-based tract images after normalizing individual tracts to the MNI space. From the group-based tract images (Fig. 3*A*), the inferior CST close to the brainstem showed high consistency across participants and the superior CST had large individual variability when it approaches the cortex. Similarly, there was a large tract variability where the OR approaches V1 (Fig. 4*A*).

We calculated the weighted NODDI (ODI, NDI) and weighted DTI (FA, MD) metrics from the 30 segments of each tract, equidistant along the tract's principal skeleton (Figs. 3*C*, 4*C*). A repeated-measures ANOVA on microstructural metrics showed significant main effects of segments in CST (NDI: *F*_(5.04, 226.65)_ = 20.82, *p* = 3.44E−17; ODI: *F*_(4.18,188.28)_ = 550.94, *p* = 3.01E−104; FA: *F*_(5.47,246.29)_ = 730.35, *p* = 1.408E−149; MD: *F*_(5.14,231.08)_ = 168.80, *p* = 5.48E−76, Greenhouse–Geisser corrected) and OR (NDI: *F*_(8.01,360.37)_ = 64.13, *p* = 9.99 E−65; ODI: *F*_(8.08,363.42)_ = 126.63, *p* = 1.1E−100; FA: *F*_(8.72,392.55)_ = 71.62, *p* = 1.01E−245; MD: *F*_(11.65,524.08)_ = 32.38, *p* = 9.75E−132).

In the CST, there was significant hemispheric difference in NDI (*F*_(1,45)_ = 18.35, *p* = 0.000095) and MD (*F*_(1,45)_ = 17, *p* = 0.00016), with lower NDI and MD values in the left hemisphere as compared with the right, consistent with previous results that CST displays a structural asymmetry independent of handedness (Hervé et al., 2006; Westerhausen et al., 2007; Powell et al., 2012; Seizeur et al., 2014; Andersen and Siebner, 2018). The hemispheric difference in ODI and FA did not reach significance (ODI: *F*_(1,45)_ = 3.81, *p* = 0.057; FA: *F*_(1,45)_ = 1.85, *p* = 0.18). In the OR, there was significant hemispheric difference in all metrics (NDI: *F*_(1,45)_ = 61.18, *p* = 6.27E−10; ODI: *F*_(1,45)_ = 34.89, *p* = 4.29E−7; FA: *F*_(1,45)_ = 15.22, *p* = 0.00032; MD: *F*_(1,45)_ = 6.7, *p* = 0.013).

These main effects were qualified by significant interactions between segment locations and hemispheres in CST (NDI: *F*_(6.04,271.79)_ = 3.569, *p* = 0.002; ODI: *F*_(6.43,289.33)_ = 20.13, *p* = 1.15E−20; FA: *F*_(8.78,395.14)_ = 23.41, *p* = 1.98E−31; MD: *F*_(9.15,411.59)_ = 4.4, *p* = 0.000014) and OR (NDI: *F*_(9.55,429.75)_ = 17.862, *p* = 4.134E−75; ODI: *F*_(10.39,467.53)_ = 9.72, *p* = 2.995E−15; FA: *F*_(9.43,424.34)_ = 3.8, *p* = 0.000095; MD: *F*_(11.43,514.51)_ = 4.76, *p* = 3.59E−7). Therefore, there were substantial variations in microstructural metrics along CST and OR consistent with other studies (Yeatman et al., 2012; Johnson et al., 2014).

We examined the correlations between the microstructural metrics along tract segments. TFCE with 10,000 permutations was used to correct multiple comparisons for the number of segments along tracts. NDI was positively correlated with ODI in superior segments of the CST (left CST: segments 23–30; right CST: segments 25–30, *p* < 0.05 TFCE-corrected) and anterior segments of OR (left OR: segments 20–29; right OR: segments 18–28, *p* < 0.05 TFCE-corrected). Consistent with previous results of NODDI measures and their relationship with DTI metrics (H. Zhang et al., 2012), ODI was negatively correlated with FA (left CST: segments 1–30; right CST: segments 1–30; left OR: segments 1–30, right OR: segments 1–30, *p* < 0.05 TFCE-corrected) and MD (left CST: segments 1–17 and 19–30; right CST: segments 3–10, 12 and 16–30; left OR: segments 19–30, right OR: segments 20–30, *p* < 0.05 TFCE-corrected).

### Correlating response speed measures with tract microstructure

We used general linear models to examine the associations between response speed measures (RT and *T*_er_) and microstructure metrics in all tract segments, including age as a nuisance variable. TFCE with 10,000 permutations was used to correct multiple comparisons for the number of segments along tracts and across all microstructural metrics (NDI, DI, FA, and MD).

**Figure 5.**
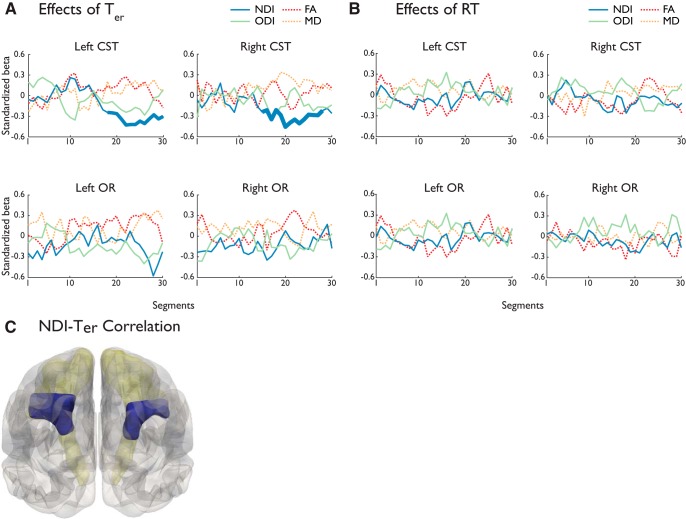
Standardized linear regression coefficients between microstructural metrics and *T*_er_ (***A***) and mean RT (***B***). The segments with significant associations after TFCE (*p* < 0.05; 10,000 permutations) are presented with increased line thickness. ***C***, CST segments that had significant negative NDI-*T*_er_ correlations were highlighted (blue) over the group-based tract volume.

For the CST (Fig. 5*A*), faster *T*_er_ was associated with higher NDI values in the superior segments of both left (segments 18–30, *p* < 0.05, TFCE-corrected) and right tracts (segments 15–28, *p* < 0.05, TFCE-corrected). The segments with significant correlations comprised the posterior limb of interior capsule and the SCR that connect to the precentral gyrus (Fig. 5*C*). No other microstructural metrics of the CST had significant correlation with the *T*_er_ (across all segments of the left CST: *p* > 0.323; right CST: *p* > 0.116).

For the OR (Fig. 5*A*), there was no significant correlation between *T*_er_ and any microstructural metrics after correction of multiple comparison (across all segments of the left OR: *p* > 0.076; right OR: *p* > 0.24).

There was no significant correlation between mean RT and microstructural metrics in CST (across all segments of the left CST: *p* > 0.425; right CST: *p* > 0.31) or OR (across all segments of the left OR: *p* > 0.108; right OR: *p* > 0.479; Fig. 5*B*).

### Specificity analysis: comparison white-matter tract and model parameters

Is it possible that the significant structural-functional associations we observed could simply be a global whole-brain white-matter property (Penke et al., 2010; Johnson et al., 2015)? To address this possibility, we have included the analysis of the bilateral dorsal CB as a comparison tract. As a part of the limbic system, the CB is important to emotional processing, memory and social behavior (Bubb et al., 2018), but we do not anticipate microstructural variations in this tract to be associated with the non-decision time in the simple RT task. We applied the same analysis pipeline on bilateral dorsal CB. There was no significant correlation between *T_er_* and any microstructural metrics (across all segments of the left CB: *p* > 0.43; right CB: *p* > 0.249, TFCE-corrected). Similarly, we did not observe any significant correlation between mean RT and microstructural metrics (across all segments of the left CB: *p* > 0.14; right CB: *p* > 0.157).

Although the current study had a priori hypotheses for correlations between *T*_er_ and microstructural metrics in CST and OR, previous research showed that, in choice RT tasks, the decision threshold correlated with tract strength between pre-SMA and subthalamic nucleus (Forstmann et al., 2010, 2011). We therefore examined correlations between other LBA model parameters (threshold *B* and mean drift rate μ) and microstructural metrics in an exploratory analysis. This analysis showed no significant result for any microstructural metrics in CST (across all segments, μ: *p* > 0.13; *B*: *p* > 0.186) or OR (across all segments, μ: *p* > 0.571; *B*: *p* > 0.17).

## Discussion

By combining cognitive and biophysical models with along-tract analyses, we found that the NDI of superior segments of bilateral CST negatively correlated with the non-decision time (*T*_er_), a model-derived component of RT that represents the duration of non-decision processes (Donkin et al., 2011).

NODDI quantifies intracellular neurite density separately from orientation dispersion (H. Zhang et al., 2012), which are otherwise indistinguishable in standard DTI measures. NODDI measures have been shown to be sensitive to microstructural changes in brain development (Genc et al., 2018), psychosis (Rae et al., 2017), and neurodegeneration (Colgan et al., 2016). The CST segments with negative NDI-*T*_er_ correlations included a part of the SCR and its connections to the precentral gyrus. This region had a large dispersion of fiber orientation, as indicated by the large ODI values (Fig. 3*C*). Therefore, a simple tensor model would be less sensitive to detection of effects with behavioral measures, highlighting the advantages of compartment models such as NODDI (Groeschel et al., 2016). The NDI-*T*_er_ correlation observed in this region may relate to somatotopic organization of the CST (Seo et al., 2012) and distal (finger) movements used in the task, which needs to be confirmed in future studies combining functional localizers and tractography (Dalamagkas et al., 2019).

In both humans (Fukutomi et al., 2018) and rodents (Jespersen et al., 2010; Sepehrband et al., 2015), neurite density estimates are sensitive to myelin content and packing density, which may affect the axonal conduction delay in white-matter tracts across individuals (Waxman, 1980; Fields, 2008; Seidl, 2014; McDougall et al., 2018). However, the conduction delay between neural activity in the motor cortex and electromyographic signals is only 10∼30 ms (Gross et al., 2000; Riddle and Baker, 2005; Volz et al., 2015), too short to be a dominant component of *T_er_*, and the properties of the gray-matter circuitry cannot be ignored. It has been shown that even a subtle change in conduction delay can have profound impacts on spike timing and oscillatory coupling over long-range connections (Pajevic et al., 2014) that are necessary for motor control (Baker et al., 2003; Pogosyan et al., 2009). Our results suggested that participants with faster motor speed may have a more efficient corticospinal network for voluntary actions (Michaels et al., 2015), with its white-matter properties reflected in higher NDI values in CST.

Electrophysiological evidence is consistent with this hypothesis. Beta-band oscillation in sensorimotor cortices have high intraindividual stability (Espenhahn et al., 2017), and beta-band corticomuscular coherence relates to visuomotor performance (Kristeva et al., 2007), suggesting a functional role of synchronized information transmission between cortex activity and electromyography (Baker, 2007). This corticospinal transmission is interrupted in ALS, evident by axonal loss (Smith, 1960) and reduced NDI (Broad et al., 2019) in the CST, and ALS patients exhibit impaired cortical oscillation and corticomuscular coherence with prolonged RT (Proudfoot et al., 2017). Microstructural efficiency and neural synchrony are not independent but rather coupled tightly with complex interactions (Pajevic et al., 2014; Bells et al., 2017). To confirm that the conduction delay influences RT via modulations of oscillatory couplings in healthy individuals (Price et al., 2017), future studies need to combine white-matter microstructural measures with oscillatory signals in the gray-matter (e.g., magnetoencephalography).

We did not find a significant correlation between microstructure and behavioral measures in the OR (cf. Tuch et al., 2005). The SRT task in the current study may not be sensitive to the interindividual variability in the visual processing pathway, as suggested by the lack of change of visual event-related potential in the SRT task (Mangun and Hillyard, 1991).

Our study provides new methods for studying brain, behavior and cognition relationships. First, we used microstructural measures weighted by connection probability and along-tract analysis based on volumetric skeletonization and segmentation. Similar to previous studies (Yeatman et al., 2012), we showed significant variations in all microstructural measures along CST and OR. These variations may be because of changes in microscopic tissue properties (Murray and Coulter, 1981), local tract geometry, or neighboring environments such as partial volumes and crossing fibers. Correlations with behavioral measures were observed only in a portion along the tracts, confirming the needs to take into account microstructural variations along tracts (Johnson et al., 2014; Yeatman et al., 2014). Several methods have previously been proposed for characterizing microstructural metrics along tracts, including medial axis representations (Yushkevich et al., 2008), *b*-spline resampling of streamlines (Colby et al., 2012), arc length matching to prototype fibers (O'Donnell et al., 2009) and centroid fibers based on the minimum (Wang et al., 2016) or mean (Yeatman et al., 2012) distance of fibers within a tract.

The current study coregistered individual tractography results to a template space and generated group-level tract probability maps (Hua et al., 2008), from which the tract skeleton and equidistant segments were calculated for subsequent along-tract analysis. In both CST and OR, voxels close to the tract skeleton had high tract probabilities in the group maps, suggesting a good agreement across participants. This approach allows reduction of the noise from individual tractography and generates representative volumetric tract profiles. Nevertheless, normalization errors and variances of tract geometries may hamper the precision of inference at the individual level. To address this issue, we transformed along-tract segments back to the native space and accounted for individual heterogeneity using masks from individual tissue probability and tractography. Furthermore, we capitalized on the connection distributions from probabilistic tractography to down-weight the contributions from voxels with high noise or low certainty in the calculation of microstructural measures (Olvet et al., 2016). As a result, our method combined group-level along-tract profiling as well as individual-level fiber tracking.

Our automated analysis pipeline can be applied to other tracts. We note that, like other methods for along-tract analysis, further tests are needed to ensure the robustness in smaller tracts with more substantial variability than demonstrated here, which is beyond the scope of the current study. To facilitate future research, we have made our analysis scripts open source and freely available.

Second, our study highlighted the benefits of computational modeling of behavioral data (Forstmann et al., 2016). The LBA model decomposed RT distributions into the duration of an evidence accumulation process (Gold and Shadlen, 2007) and non-decision time *T*_er_. Individual differences in *T*_er_ are likely subject to influences at multiple processing levels. Along CST, we found that microstructural metrics correlated with the *T*_er_ but not mean RT. Considering the predominate role of CST in transmitting motor commands (Lemon, 2008; Murray et al., 2017), our results provide anatomical evidence to support the common assumption that the *T*_er_ includes motor latencies. The *T*_er_ is a unitary estimate and hence cannot distinguish between visual and motor latencies. It is possible to combine our approach with other imaging modalities to dissociate the *T*_er_ into subcomponents that occur before and after decisions (White et al., 2014; Nunez et al., 2019).

There are several limitations to this study. Our experimental samples included only female participants with a narrow age range. Both gender (Maccoby, 1991; Dykiert et al., 2012) and age (Wilkinson and Allison, 1989) have been shown to influence RT (Yang et al., 2015) and microstructural measures (Good et al., 2001; Toosy et al., 2004; Lebel et al., 2012; Bede et al., 2014; Cox et al., 2016; Kodiweera et al., 2016). On the other hand, our homogenous sample also ensured that gender and age could not be confounds that mask the influence of other variables, and hence improved sensitivity and interpretability. Further research could extend our results to heterogeneous or genetically-informed samples, because both white-matter microstructure and RT are affected by genetic and environmental factors (Martin et al., 2004; Chiang et al., 2011; Rüber et al., 2015).

We focused on two a priori tracts of primary motor and visual pathways: CST and OR, because the latencies of motor initiation and stimulus encoding are commonly assumed to comprise the *T*_er_. This does not rule out the possibility that RT in other tasks associate with microstructural measures in different white-matter tracts. Indeed, the RT in a visual oddball task correlated with the MD in tracts connecting the visual cortex with frontal and temporal cortices (Konrad et al., 2009), and the RT in a selective attention task correlated with the FA in the superior and inferior longitudinal fasciculus (Mayer and Vuong, 2014). The current study only estimated the *T*_er_ and mean RT from a single SRT task, and hence cannot infer the extent to which the microstructural correlates of response speed may vary as a function of different tasks. An important future direction is to have a mechanistic understanding of how tissue microstructure may selectively influence behavioral performance across cognitive domains.

In conclusion, interindividual differences in non-decision time during simple actions were reflected in the extent of neurite density in white-matter tracts responsible for motor information transmission. These findings help validate the functional origin of non-decision time assumed in current models of decision-making and action selection. Our results further raise an intriguing possibility that tissue microstructure in key fiber tracts importantly influences response speed in basic cognitive processes.
